# Community’s perceptions of pre-eclampsia and eclampsia in Sindh Pakistan: a qualitative study

**DOI:** 10.1186/s12978-016-0136-x

**Published:** 2016-06-08

**Authors:** Asif Raza Khowaja, Rahat Najam Qureshi, Sana Sheikh, Shujaat Zaidi, Rehana Salam, Diane Sawchuck, Marianne Vidler, Peter von Dadelszen, Zulfiqar Bhutta

**Affiliations:** Division of Women and Child Health, Aga Khan University, Karachi, Pakistan; Department of Obstetrics and Gynecology, Child and Family Research Institute, University of British Columbia, Vancouver, Canada

## Abstract

**Background:**

Maternal mortality is of global public health concern and >99 % of maternal deaths occur in less developed countries. The common causes of direct maternal death are hemorrhage, sepsis and pre-eclampsia/eclampsia. In Pakistan, pre-eclampsia/eclampsia deaths represents one-third of maternal deaths reported at the tertiary care hospital settings. This study explored community perceptions, and traditional management practices about pre-eclampsia/eclampsia.

**Methods:**

A qualitative study was conducted in Sindh Province of Pakistan from February to July 2012. Twenty-six focus groups were conducted, 19 with women of reproductive age/mothers-in-law (*N* = 173); and 7 with husbands/fathers-in-law (*N* = 65). The data were transcribed verbatim in Sindhi and Urdu, then analyzed for emerging themes and sub-themes using NVivo version 10 software.

**Results:**

Pre-eclampsia in pregnancy was not recognized as a disease and there was no name in the local languages to describe this. Women however, knew about high blood pressure and were aware they can develop it during pregnancy. It was widely believed that stress and weakness caused high blood pressure in pregnancy and it caused symptoms of headache. The perception of high blood pressure was not based on measurement but on symptoms. Self-medication was often used for headaches associated with high blood pressure. They were also awareness that severely high blood pressure could result in death.

**Conclusions:**

Community-based participatory health education strategies are recommended to dispel myths and misperceptions regarding pre-eclampsia and eclampsia. The educational initiatives should include information on the presentation, progression of illness, danger signs associated with pregnancy, and appropriate treatment.

## Background

The hypertensive disorders of pregnancy (HDP), particularly pre-eclampsia and eclampsia are among the top three leading causes of maternal mortality globally [1, 2]. Pre-eclampsia is commonly defined as the presence of new hypertension and significant proteinuria during pregnancy [3, 4]. The trajectory of the disease can put women at high risk of eclampsia- a serious condition clinically manifested by seizures in the absence of early identification and appropriate management [3]. Globally, it is estimated that HDP complicates ten million pregnancies, resulting in 70,000 to 80,000 maternal and 500,000 perinatal deaths annually [5]. A landscape analysis revealed the risk of developing pre-eclampsia is seven times greater for women in less developed countries as compared to developed countries [5]. Another study from less developed countries reported the odds of a woman dying from pre-eclampsia and eclampsia is 300 times higher than that for a woman in more developed countries [6].

The secondary analysis of the World Health Organization Global Survey on maternal and perinatal health revealed socio-demographic variables (i.e., maternal age > 30 years, and low education attainment), as well as, clinical variables (i.e., chronic hypertension, obesity and severe anemia) as the highest risk factors for pre-eclampsia in low-and-middle income countries [7].

Pakistan is the sixth most populous country in the world. A recent systematic analysis of global mortality ranked Pakistan as the country with the third highest burden of maternal, fetal and child mortality [8, 9]. According to the Pakistan Demographic and Health Survey 2006–07, the maternal mortality ratio (MMR) was 279 per 100,000 live births [10]. There are wide variations between rural and urban populations; the MMR for Sindh province (which is predominantly rural) was as high as 345–350 per 100,000 live births [11].

Eclampsia is responsible for 34 % of maternal deaths in women admitted to a tertiary care hospital in Pakistan [12]. Clinical management of pre-eclampsia and eclampsia requires hospitalization; therefore, the magnitude of pre-eclampsia-and eclampsia-related mortality may be higher in rural populations, where there are key barriers to care seeking [13, 14]. Previous studies have looked at the prevalence [15], risk factors [16], and clinical management of women with pre-eclampsia and eclampsia in Pakistan [17]. Awareness of the cultural aspects is imperative to understand women and their families’ perspective about pre-eclampsia.

This study was conducted as part of the formative research of a large community-based research trial - Community Level Interventions for Pre-eclampsia (CLIP Trial) in Sindh province, Pakistan (NCT01911494) [18]. The objective of this study was to explore the community’s understanding of pre-eclampsia and eclampsia; including local terminology, perceived causes, danger signs, prevention strategies, outcomes, and traditional treatments in the rural settings. The results of this study identify knowledge gaps at community level and can inform development of future communication strategies.

## Methods

A qualitative study was undertaken between February to July 2012, as part of a large multi-country study, the detailed methods are described elsewhere [19]. This study was conducted in Sindh- the third largest province by area in Pakistan. Two southern districts namely Hyderabad and Matiari were selected. The semi-urban district Hyderabad is located on the east bank of the Indus River, and is the second largest city of Sindh province with a population of over 1 million [20]. Matiari is rural district located 25 kilometers north of Hyderabad with a population of roughly 0.6 million [21]. Over 90 % of residents are Muslims, Sindhi and Urdu are the main dialects. The literacy rates as compared to other provinces are low (43 % for female and 67 % for male); and the major industry is agriculture.

Data were collected through focus group discussions (FGDs) [22], with women of reproductive age (15–49 years), mothers-in-law, husbands, and fathers-in-law. Other studies relevant to maternal health in the rural settings in Pakistan [12] and elsewhere [13], found that men had a key role in decisions pertaining to care seeking. Hence, husbands and fathers-in-law were included in this study to understand their perceptions and belief regarding pre-eclampsia and its prevention and management.

The FGD guides were translated into Sindhi and Urdu languages, and pilot tested for comprehension, cultural sensitivity, and duration. To respect local preferences of participants, FGDs were held separately for women and men at local venues. Data saturation was reached through 26 FGDs [23].

**Fig. 1:**
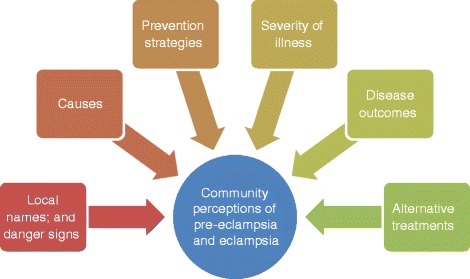
Emerging themes from the focus groups discussions

All discussions were transcribed into Sindhi and Urdu languages. Stringent data quality control measures were followed. These included random observations of FGDs, 20 % (audit-trial) verification of the content of manual transcripts by audio-recording review, and fortnightly debriefing sessions with moderators and transcribers. In addition, the moderators recoded a self-reflection after each session to describe their thoughts and impressions to better contextualize the data, as well as, to protect against self-bias. A thematic analysis (combining an inductive and deductive approach) was used with the assistance of NVivo version 10 software [QSR, Doncaster Vic, Australia]. All responses were coded to relevant nodes, which were later categorized into hierarchy of tree-nodes.Subsequently, emerging themes and sub-themes were drawn from the tree nodes (Fig. 1).

This study received ethical approval from Ethics Review Committee of Aga Khan University (1917-OBS-ERC-11), Karachi Pakistan, National Bioethics Committee of Pakistan and Clinical Research Ethics Board of University of British Columbia, Vancouver Canada (H12-00132).

## Results

Twenty-six focus groups were conducted: 19 with women of reproductive age/mothers-in-law; and 7 with husbands/fathers-in-law (Table 1).

**Table 1:** Site specific distribution of focus group discussions

Groups	Matiari	Hyderabad	Group specific total
	# of FGDs (n, number of participants)	# of FGDs (n, number of participants)	
Women of reproductive age and mothers in-law	10 (89)	09 (84)	19 (173)
Husbands and fathers-in-law	05 (49)	02 (16)	07 (65)
Site specific total	15 (138)	11 (100)	26 (238)

The mean age of female participants was 28.5 years, 89 % were housewives, and 49 % never attended school. The mean age of male participants was 35 years, 92 % were self-employed in the agriculture sector, and 37 % had never attended school.

### Local names and danger signs

A large number of participants, both women and men, had no knowledge of a condition such as pre-eclampsia or eclampsia associated with pregnancy. When asked a direct question if they knew about blood pressure they answered in the affirmative. They believed that even women with normal blood pressure before pregnancy may experience hypertension during pregnancy. Participants used ‘*Rat jho dabao vadhan*’ [Sindhi] to express their understanding of ‘*high blood pressure*’. Women were asked about symptoms of high blood pressure and they so reported ‘*mathay-mein-soor’, ‘chakar’, ‘ulti-wanghar-mehsoos-thiyan’,*and *‘kamzoori’* [Sindhi] to represent hypertension in pregnancy. These terms can be translated as headache, dizziness, nausea/vomiting, and weaknesses; all of which are general symptoms associated with pregnancy. The women’s perception about symptoms suggestive of high blood pressure was not related with objective testing of the blood pressure of the women. Women and men reported that they knew about seizures and used the words ‘*jhatka*’ [English translation: *fits*] by majority of participants. Seizures were reported as [in Sindhi] ‘*khatarnak alaamat*’ [English translation: *danger sign*] for mothers and newborns. They were not aware of the association of high blood pressure with seizures.

### Perceived causes

Most participants mentioned that [Sindhi] ‘*ghano-soochanr*’, ‘*pareshani*’ [English translation: *excessive thinking* or *stress]* are the most common causes of high blood pressure in pregnancy. Participants ascribed lack of rest, domestic problems [in Sindhi] ‘gharelo-pareshani’, the burdens of household chores and social responsibilities, as the reasons for maternal stress in pregnancy. One woman described the following:

“Blood pressure increases during pregnancy only because of excessive thinking, mental stress and tension about children”.

Participant 3, FGD 4, woman of reproductive age

A few participants reported early marriages, anemia and low blood sugar levels as some of the other causes of stress to the mother. A large majority of participants believed that weakness, anemia, and maternal stress were the leading causes of seizures during pregnancy. A few also considered that seizures were supernatural in origin.

### Prevention strategies

The predominant perception in this community was that hypertension and seizures in pregnancy were the result of maternal stress; therefore, families should provide support to alleviate stress.

While discussing the roles of household decision makers many women had good relationship with mothers-in-law by receiving emotional support, sharing of household chores, getting help with childcare, and accompanied to health facility in case of emergency. Whereas, the roles of husband and father-in-law were perceived to be more as facilitators: to provide permission, to arrange transport, and to provide financial support. One male decision-maker described this in the following quote:

“The role of husband is critical! After all, the mother and baby are his responsibility. Therefore, it depends on the way he treats his wife, feeds her well, and gives her respect”.

Participant 4, FGD 3, male decision-maker

Many also recommended a healthy diet rich in fat, and adequate rest to prevent hypertension and seizures during pregnancy.

Some women complained of their mother-in-law’s insensitivity to her problems in pregnancy. A woman described this in the following quote:

“When [she is] sick, [her] mother-in-law thinks that [she is] too weak. She often criticizes and says…don’t think, you are the only one pregnant, and no other women have delivered before you”.

Participant 1, FGD 6, woman of reproductive age

### Perceived severity

The severity of hypertension during pregnancy was mainly recognized with aggravated signs and symptoms. Participants discussed how pregnant woman experienced an ‘*increasing intensity of headache with dizziness*’; ‘*inability to do household chores*’; ‘*feelings of severe weaknesses’;* and an *‘altered level of consciousness’.*

One-male participant described the severity of hypertension in the following quote:

“She gets seriously ill, [and] cannot work in the house at all”.

Participant 9, FGD 2, male decision-maker

Seizures during pregnancy were perceived to be ‘*a sign for health emergency’*. Many participants mentioned that women must be taken to the health facility, in case of seizures.

### Perceived outcomes

All participants believed that hypertension and seizures during pregnancy increased the risk of poor pregnancy outcomes. The main consequences of hypertension and seizures as reported by participants included complications during labor, death of mother, stillbirth, and weakness of the newborn and low birth weight. Likewise, a woman participant revealed as quoted below:

“When mother’s blood pressure rises, the vein of the brain can rupture. It can kill the mother and the baby in womb”.

Participant 1, FGD 1, woman of reproductive age

Many participants revealed that seizures during pregnancy could lead to unconsciousness, which may result in death. Only a few mentioned post-pregnancy complication of hypertension and seizures, such as delayed development of the baby. A male participant explained this in the following quote:

“If mother is affected by increased pressure and seizures, naturally it will affect baby’s health after birth”.

Participant 5, FDG 7, male decision-maker

### Alternative treatments

Almost all participants reported self-medication for symptoms, such as headache. Although participants did not recall the name of the medication used for managing hypertension during pregnancy, they mentioned [in Sindhi] ‘*Soor-ji-dawa*’ [English translation: *pain killers]*, which were commonly available without prescription. The use of other home remedies, spiritual treatments and alternative medicines were not commonly reported for managing hypertension and seizures during pregnancy. Only a few women of reproductive age [in a rural district] believed in traditional treatments such as ‘*reciting holy verses, asking mother to drink holy water, [and] massaging with coconut oil*’ could be beneficial. Few male decision-makers, also from a rural district, believed home remedies and spiritual treatments could reduce the severity of blood pressure, and manage seizures.

## Discussion

Despite substantial global investments to reduce maternal mortality over the past decade, many countries in Sub-Saharan Africa and South Asia have made slow progress towards Millennium Development Goal-5 [24]. Cause-specific maternal mortality from pre-eclampsia and eclampsia, albeit alarmingly high, has received insufficient attention in health policy context [25]. In particular early recognition and management at the community level, where many women are dying in less developed countries, has largely been omitted from recent research initiatives [25].

This study contributed to community understandings about pre-eclampsia and eclampsia. These community beliefs included misperceptions regarding danger signs, underlying causes, prevention strategies, outcomes, and management. The literature suggests that the HDP are commonly misunderstood in less developed countries due to illiteracy, lack of awareness, superstitious beliefs, and poverty [26, 27]. Both high blood pressure and seizures were often perceived to be potentially dangerous for mother and baby. Therefore, it is clear that practices for disease prevention and traditional management of pre-eclampsia and eclampsia were deeply rooted in perceptions of disease. Literature also suggests about the delay in recognition of severity, which results in large number of maternal deaths at home or on the way to health facility that could otherwise be averted [28, 29].

Self-medication was reported, as the first choice of treatment for severe headache during pregnancy. Our findings are corroborated with another recent study that also suggested increasing trend of self-medication in Pakistan. It was reported that easy access to over the counter medication and prescription-only medication are main determinant for self-medication in the country [30]. However, such contrary practices of self-medication may have serious consequences particularly for pregnant women, who are at risk of developing lethal complications, due to delay of appropriate treatment [31].

### Strengths and limitations

This study bridges the knowledge gap for community perceptions surrounding pre-eclampsia and eclampsia in Pakistan. FGDs were scheduled at convenient times and venues to accommodate participants with minimal distractions. Separate sessions for household decision makers (husbands, fathers-in-law, mothers-in-law) were not possible given logistic and resource limitations; as a result, it is possible that some participants did not feel comfortable to actively participate despite encouragement by the moderator. Combined focus groups were held for women of reproductive age and mothers-in-law, therefore some women were hesitant to speak-up. Similarly, group sessions for husbands and fathers-in-law were combined. The combined focus groups may have impeded open dialogue because potential cultural barrier whereby young people are unlikely to oppose senior members of the community.

## Conclusions

This qualitative study provides insights of community’s understanding of pre-eclampsia ascribed as general symptoms, and less specific to clinical conditions. There were mixed opinions regarding the causes of the hypertension in pregnancy and a poor understanding regarding the connection between pregnancy, hypertension and seizures.

Community-based participatory health education strategies are highly recommended to address myths and misperceptions about danger signs of pregnancy in Pakistan. Dissemination of knowledge to the wider community would likely challenge traditional beliefs, customs and practices; therefore behavior change communication would be very useful strategies to implement at community settings. Education should be integrated into training programs for community health workers to improve their knowledge base and facilitate community awareness in rural Pakistan.

## Abbreviations

HDP: hypertensive disorders of pregnancy
MMR: maternal mortality ratio
CLIP: community level interventions for pre-eclampsia
FGD: focus group discussion
